# Post-cardiopulmonary bypass colloid osmotic pressure and creatinine-defined acute kidney injury in infants undergoing cardiac surgery: an age-modified association

**DOI:** 10.3389/fped.2026.1929104

**Published:** 2026-10-14

**Authors:** Yuan Zhou, Wenting Gui, Xiaoyu Zhang, Jiaxiang Chen, Jiahui Xie, Kai Liu, Song Yi, Runwei Ma

**Affiliations:** 1 Department of Cardiac Surgery, Fuwai Yunnan Hospital, Chinese Academy of Medical Sciences/Affiliated Cardiovascular Hospital of Kunming Medical University, Kunming, Yunnan, China; 2 Department of Cardiopulmonary Bypass, Fuwai Yunnan Hospital, Chinese Academy of Medical Sciences/Affiliated Cardiovascular Hospital of Kunming Medical University, Kunming, Yunnan, China; 3 Department of Comprehensive Pediatrics, Kunming Children's Hospital, Kunming, Yunnan, China

**Keywords:** acute kidney injury, cardiopulmonary bypass, colloid osmotic pressure, generalized additive model, infants, perioperative risk marker

## Abstract

**Background:**

Acute kidney injury (AKI) is common after infant cardiac surgery with cardiopulmonary bypass (CPB). Colloid osmotic pressure (COP) changes during CPB in response to hemodilution, albumin administration, ultrafiltration, and hemoconcentration, but its association with postoperative AKI in infants remains incompletely defined. Because renal physiology matures rapidly during the first year of life, the clinical meaning of COP may also vary across infancy.

**Methods:**

We conducted a single-center retrospective cohort study of 230 infants aged ≤12 months and weighing ≤10 kg who underwent cardiac surgery with CPB between May 2020 and September 2022. The primary exposure was COP measured immediately after CPB, and the primary outcome was creatinine-defined postoperative AKI based on KDIGO thresholds within a prespecified 48-hour postoperative window. Multivariable logistic regression evaluated the covariate-adjusted association between post-CPB COP and AKI. Restricted cubic splines assessed dose-response form, and a generalized additive model (GAM) with a tensor-product smooth of COP and age was used to explore age-related heterogeneity. Time-specific analyses additionally evaluated pre-CPB, intra-CPB, and post-CPB COP.

**Results:**

AKI occurred in 57 infants (24.8%). After adjustment for covariates, for every 1 mmHg increase in post-CPB COP, the risk of AKI increased by 16% (OR = 1.16; 95% CI: 1.04–1.28; *P* = 0.004). The dose–response relationship was broadly linear. The GAM curve combining age and COP suggested age-related heterogeneity (*P* = 0.022); in an exploratory age-stratified analysis, the estimated odds ratio was highest in the group of infants aged 10–12 months (OR per 1 mmHg increase: 1.79; 95% CI: 1.09–3.64; *P* = 0.048); however, due to the small number of events in this subgroup, these findings should be interpreted as exploratory. There was no significant association between pre-CPB or during-CPB COP and AKI, whereas post-CPB COP exhibited the strongest time-specific signal. Incorporating COP into the clinical model resulted in only a slight, non-statistically significant improvement in discrimination performance (AUC: 0.701 vs. 0.723; *P* = 0.324); no significant improvement was observed in NRI, and the overall net benefit was minimal.

**Conclusions:**

Higher post-CPB COP was associated with creatinine-defined AKI after infant cardiac surgery, with the clearest risk information observed at the end of CPB. COP is better interpreted as a peri-CPB physiologic risk marker that should be considered alongside age and perfusion context, rather than as an independent prediction tool or a fixed therapeutic target.

## 1 Introduction

Cardiac surgery during infancy remains a high-risk setting in which perioperative organ protection is central to postoperative recovery. Acute kidney injury (AKI) is among the most frequent complications after pediatric cardiac surgery; pooled estimates suggest that approximately one third of children develop AKI, although incidence varies with case mix and diagnostic criteria (1, 2). Postoperative AKI is associated with prolonged mechanical ventilation, longer intensive care and hospital stays, greater need for renal replacement therapy, and increased short-term morbidity (3). Evidence regarding long-term kidney outcomes is more heterogeneous, but persistent renal abnormalities have been reported in a subset of survivors, reinforcing the importance of perioperative kidney protection (4).

Renal physiology changes rapidly throughout the first year of life. Glomerular filtration rate (GFR) is low at birth, rises steeply during early postnatal adaptation as renal blood flow increases and renal vascular resistance falls, and continues to mature throughout infancy (5, 6). Tubular sodium and water handling, urinary concentrating capacity, acid-base regulation, transporter expression, and autoregulatory responses also mature after birth, but not necessarily in parallel with GFR (7). Prematurity introduces additional heterogeneity because nephrogenesis normally continues into late gestation; infants of the same chronological age may therefore differ in renal developmental maturity (6). These developmental features may modify renal responses to CPB-related changes in perfusion pressure, oxygen delivery, hemodilution, inflammation, and fluid management.

Colloid osmotic pressure (COP), or oncotic pressure, is the osmotic pressure generated by plasma macromolecules that are relatively retained by the microvascular barrier. Albumin is its principal plasma determinant, although globulins and exogenous colloids also contribute; COP therefore cannot be regarded as interchangeable with serum albumin concentration (8). Under the revised Starling framework, the influence of COP on transvascular fluid exchange also depends on hydrostatic pressure, endothelial glycocalyx integrity, the protein reflection coefficient, and microvascular permeability (8, 9). During CPB, prime-related hemodilution can lower plasma protein concentration and COP, whereas albumin-containing prime, albumin administration, and hemoconcentration produced by conventional or modified ultrafiltration may subsequently increase COP. Pediatric studies have examined albumin-containing CPB prime and ultrafiltration as components of perfusion management, but their renal implications remain uncertain (10, 11). Thus, peri-CPB COP may represent an integrated physiologic measure of plasma protein composition, hemodilution, ultrafiltration, and colloid management rather than a simple marker of intravascular volume.

Importantly, a higher COP should not automatically be interpreted as beneficial, and COP itself should not be equated with blood viscosity. A higher post-CPB COP may accompany hemoconcentration and increased plasma protein concentration, changes that can also affect plasma rheology. In adult cardiac surgery, plasma viscosity varies across the perioperative period and is strongly related to plasma protein concentration (12). CPB may also impair endothelial and glycocalyx integrity, alter microvascular reactivity, and produce heterogeneous tissue perfusion, while renal medullary hypoxia and inflammation are recognized components of CPB-associated kidney injury (13, 14). Pediatric data further link lower indexed oxygen delivery during CPB with postoperative AKI (15). These observations raise the possibility that higher post-CPB COP identifies a broader hemoconcentration-perfusion phenotype rather than acting as an isolated nephrotoxic exposure.

Most studies of pediatric cardiac surgery-associated AKI have focused on age, surgical complexity, CPB duration, transfusion, fluid overload, and hemodynamic or oxygen-delivery disturbances. Directly measured COP and its temporal behavior across CPB have received far less attention. We therefore examined the association between directly measured post-CPB COP and creatinine-defined postoperative AKI in infants undergoing cardiac surgery. Post-CPB COP was prespecified as the primary exposure, while available pre-CPB and intra-CPB measurements were used to place this exposure in its peri-CPB temporal context. We further evaluated dose-response form, explored possible age-related heterogeneity, and assessed whether COP added risk information beyond conventional clinical and perfusion variables.

## 2 Methods

### 2.1 Ethics approval

The study was approved by the Ethics Committee of Fuwai Yunnan Cardiovascular Hospital (approval No. 2023-056-01; October 27, 2023) and was conducted in accordance with the Declaration of Helsinki. Because this was a retrospective study using de-identified data, the requirement for informed consent was waived by the ethics committee.

### 2.2 Study design, setting, and population

This single-center retrospective cohort study consecutively included infants with congenital heart disease who underwent cardiac surgery with cardiopulmonary bypass (CPB) at Fuwai Yunnan Cardiovascular Hospital between May 2020 and September 2022. Infants were eligible if they were ≤12 months of age and weighed ≤10 kg at surgery. Exclusion criteria were emergency surgery, preoperative mechanical ventilation, previous cardiac surgery, and missing data for the primary exposure, particularly the absence of a post-CPB COP measurement.

The most consistently available measure of preoperative renal status was the most recent serum creatinine value within 24 h before surgery. Because serial preoperative creatinine measurements and complete, time-resolved preoperative urine-output data were unavailable, pre-existing AKI could not be retrospectively adjudicated for all infants using full KDIGO criteria. A preoperative creatinine concentration >70 μmol/L was therefore used only as an operational definition of preoperative renal impairment in sensitivity analyses, not as a diagnostic criterion for preoperative AKI. Data were complete for the primary exposure, primary outcome, and covariates included in the primary model; postoperative peritoneal dialysis status was unavailable for one infant.

### 2.3 CPB management, COP assessment, and perioperative fluid management

All procedures were performed using a broadly standardized CPB strategy with a roller-pump system (LivaNova Sorin Stockert S5) and a CAPIOX FX05 oxygenator. The CPB prime generally contained Ringer acetate, 25% human albumin, 4% succinylated gelatin, and red blood cells, with composition individualized according to body weight, baseline hematocrit, procedure, and perfusion requirements. Conventional ultrafiltration (CUF) and modified ultrafiltration (MUF) were applied according to the institutional perfusion protocol; patient-level CUF and MUF volumes were extracted from perfusion records and normalized to body weight. Detailed run-level parameters such as instantaneous ultrafiltration rate and exact MUF duration were not consistently available for retrospective reconstruction. No prespecified COP treatment target was used in the study.

COP was measured directly with a BMT923 membrane osmometer (BMT MESSTECHNIK GmbH, Germany), which determines the equilibrium pressure difference generated across a semipermeable membrane. According to the institutional perfusion workflow, COP was recorded at four prespecified points: after preparation of the CPB prime, after heparinization immediately before CPB, during CPB, and immediately after CPB. The post-CPB value was prespecified as the primary exposure because it integrates the effects of hemodilution, albumin use, ultrafiltration, and hemoconcentration during CPB and was obtained before transfer to the ICU and before postoperative diuresis or fluid resuscitation. The pre-CPB measurement was the earliest patient-level COP available, but it was obtained after perioperative intravenous treatment had begun and therefore cannot be considered a true untreated preoperative baseline.

Intraoperative fluid balance was calculated as recorded total fluid input minus recorded total fluid output and was normalized to body weight (mL/kg). Inputs included crystalloids, colloids, human albumin, blood products, and intravenous medication infusions. Outputs included urinary output, CUF and MUF volumes, and estimated blood loss. Blood loss was estimated from suction-canister volume and sponge/gauze weighing. Fluid or blood losses that could not be reliably recovered from surgical drapes or other surfaces, blood drawn for laboratory testing, and insensible evaporative losses were not routinely quantified and were not included.

The fluid-balance variable covered the intraoperative period only. Early postoperative cumulative fluid balance, hourly urine output, diuretic exposure, and fluid-resuscitation data were not sufficiently complete or standardized for formal analysis. Because the primary post-CPB COP measurement was obtained before ICU transfer, subsequent postoperative fluid management could not alter the measured exposure; however, postoperative fluid shifts, diuresis, and hemodynamic support may still influence subsequent AKI and are therefore considered potential sources of residual confounding.

Blood-product exposure was recorded separately for red blood cells used in the CPB prime and for additional intraoperative packed red blood cells (PRBC) and fresh frozen plasma (FFP). Volumes were normalized to body weight (mL/kg). When source records were expressed in units, volumes were converted using the institution-specific nominal volume documented in the source dataset. Because additional PRBC and FFP transfusions were uncommon, these variables were reported descriptively rather than entered as separate covariates in the primary model.

### 2.4 Study outcome and AKI severity

The primary endpoint was postoperative AKI, defined based on the KDIGO serum creatinine threshold within a predefined 48-hour postoperative window (16). AKI was diagnosed when the peak postoperative creatinine level exceeded the preoperative baseline by ≥26.5 µmol/L or reached at least 1.5 times the baseline value within the first 48 h after surgery. Baseline creatinine was defined as the most recent measurement obtained within 24 h prior to surgery. While the standard KDIGO criterion of ≥1.5× baseline allows for such an increase to occur within 7 days, this study employed a stricter 48-hour window.

The cumulative intraoperative urine output can be utilized for fluid balance calculations; however, the continuity of perioperative continuous urine output recordings (particularly postoperative ICU-hour urine output) is often insufficient, making it impossible to reliably apply the KDIGO duration-dependent urine output criteria. This may lead to underestimation of AKI cases characterized primarily by oliguria. Similar approaches relying solely on creatinine-based criteria have been observed in previous studies of pediatric cardiac surgery (17, 18).

In patients diagnosed with AKI based on creatinine levels, Stage 1, 2, and 3 are further classified according to the KDIGO serum creatinine threshold to indicate the corresponding severity level. Since urine output and renal replacement therapy criteria were not included in the predefined outcome definitions, this classification is referred to as the “Creatinine-Defined AKI Severity Scale” rather than the complete KDIGO staging system. Postoperative peritoneal dialysis (PD) was recorded as a separate secondary clinical outcome and was not used to redefine the AKI grading based on creatinine levels.

### 2.5 Covariate selection and analytical framework

The covariates in the multivariate model were pre-defined based on clinical evidence and perioperative pathophysiological relevance, rather than being selected based on univariate *P*-values. The main model included COP after CPB, age, sex, body weight, Risk Adjustment for Congenital Heart Surgery-1 (RACHS-1) score (19), CPB duration, cardiopulmonary bypass discontinuation, intraoperative fluid balance, and albumin dosage, which respectively reflect developmental stage, body habitus, surgical risk, CPB exposure, and perioperative volume/collateral management. BMI was calculated as body weight (kg)/height (m)^2^ and was used solely for descriptive reporting purposes; it was not included in the main model alongside body weight.

The COP post-CPB is an ex post measurement influenced by prior blood dilution, fluid therapy, albumin infusion, ultrafiltration, and blood concentration; therefore, this study does not aim to estimate the causal effect of artificial modifications on the COP, but rather to evaluate whether the observed COP values within a real-world perioperative management context carry risk information associated with subsequent AKI. This study pre-defined a time-causal framework (Supplementary Figure S1): baseline patient characteristics and procedural/perfusion-related factors occurred prior to COP measurement; intraoperative fluid balance, albumin administration, and ultrafiltration served as upstream management determinants for the COP; postoperative ICU fluid management, diuretic use, and hemodynamic support occurred after the primary COP measurement and were excluded from the main model. A sensitivity analysis of the model specification further assessed the impact of adjusting for intraoperative management variables on the estimation of the COP effect.

### 2.6 Statistical analysis

Continuous variables are summarized according to their distribution using the mean ± standard deviation or the median (interquartile range); categorical variables are presented as counts (or percentages). For comparisons between groups of continuous variables, the t-test or the Mann–Whitney U test is employed; for categorical variables, the *χ*^2^ test or Fisher's exact test is used.

The association between COP and AKI was first assessed using univariate logistic regression, followed by multivariate logistic regression analysis adjusted for predefined covariates; the adjusted odds ratios (OR) and 95% confidence intervals (CI) were reported. In the primary analysis, COP was treated as a continuous variable.

To avoid making prior assumptions about a linear relationship, restricted cubic spline (RCS) regression was employed to model the dose–response curve, with nodes placed at the 10th, 50th, and 90th percentiles of the COP distribution; nonlinear components were then formally tested.

Given the continuous development of renal function during infancy, a generalized additive model (GAM) was employed to explore the joint nonlinear relationship between age and post-cardiopulmonary bypass (CPB) COP (20). The model fitted a tensor product smoothing term for COP vs. age, while adjusting for sex, body weight, RACHS-1 score, CPB duration, circulatory arrest time, fluid balance, and albumin dosage. The maximum basis dimension was set to 5; the smoothing parameter was estimated using the restricted maximum likelihood method, and the effective degrees of freedom, basis dimension adequacy, and model convergence were verified. Age-stratified logistic regression was used solely to present exploratory subgroup estimates and should not be interpreted as independent evidence of differences in effect sizes across different age groups. Time-specific analyses were conducted to evaluate the associations between COP at pre-CPB, intra-CPB, and post-CPB time points, as well as their respective change scores (from pre-CPB to post-CPB, and from intra-CPB to post-CPB), with AKI; each time point was modeled using the same adjustment framework. Additionally, the COP change scores (*Δ*COP) from pre-CPB to post-CPB and from intra-CPB to post-CPB were further calculated, and their relationships with AKI were separately assessed.

An exploratory analysis was conducted to examine the interaction term between COP and fluid balance or albumin dosage. A regression-based mediation framework combined with 1,000 bootstrap resampling was employed for descriptive statistical decomposition (21); since intraoperative fluid balance occurred prior to COP measurement after CPB, this analysis did not involve causal mediation interpretation. Incremental predictive value was assessed using the area under the receiver operating characteristic curve (AUC), and comparisons of AUC values were performed using the DeLong method (22). The Net Reclassification Improvement (NRI) index (23) and Decision Curve Analysis (DCA) (24) were used as exploratory indicators of incremental value.

The sensitivity analysis sequentially excluded patients with neonatal age (<1 month), in-hospital mortality, preoperative serum creatinine > 70 µmol/L, or extreme COP values (<10 or >22 mmHg). Other analyses included adjustments for preoperative albumin levels, exclusion of patient/s with preoperative albumin <35 g/L, restriction to non-cyanotic infants, and comparison of model specifications incorporating baseline creatinine vs. those using different perioperative management variables. Since the bypass interruption time is highly correlated with CPB duration, the bypass interruption time was used as a substitute for CPB duration in the sensitivity analysis rather than being included alongside it. Supplementary analyses were conducted to evaluate the relationship between CPB duration and total ultrafiltration volume (mL/kg), the relationship between total ultrafiltration volume and post-CPB COP, as well as to further adjust for the impact of ultrafiltration on COP-AKI estimates. Given that ultrafiltration is incorporated into the fluid balance calculations, collinearity was first assessed when models incorporating both parameters were included. The exploratory dichotomization of 16 mmHg thresholds was evaluated using inverse probability weighting (25), while robustness assessments for unmeasured confounders were summarized using E-values (26).

All statistical tests were two-sided; a *P*-value <0.05 was used as the statistical significance threshold, with effect sizes and 95% confidence intervals provided for interpretation. The analyses were performed using R software.

## 3 Results

### 3.1 Study population and baseline characteristics

Of 297 infants screened, 230 met the prespecified eligibility criteria and were included in the final analysis (Figure 1). Median age was 6.0 months (IQR, 4.0–9.0), median body weight was 6.3 kg (IQR, 5.2–7.4), and 117 infants (50.9%) were male. Fifty-seven infants (24.8%) developed postoperative creatinine-defined AKI.

**Figure 1:**
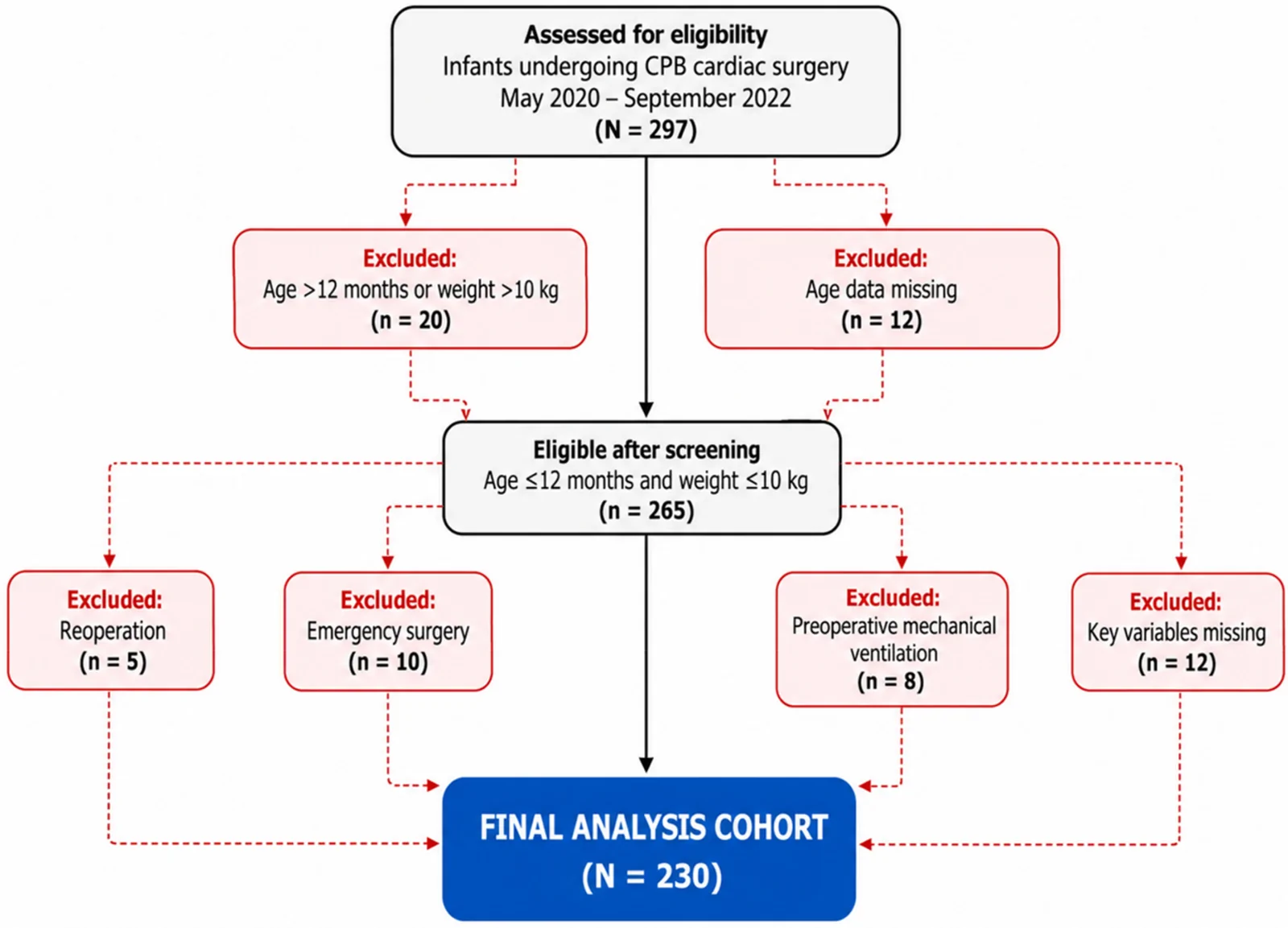
Study flow diagram.

Baseline characteristics by AKI status are summarized in Table 1. Compared with infants without AKI, those who developed AKI were younger (median, 5.0 vs. 7.0 months; *P* = 0.001), had longer CPB duration (105 vs. 91 min; *P* = 0.038), and had higher post-CPB COP (16.3 vs. 15.5 mmHg; *P* = 0.038). RACHS-1 score and cross-clamp duration were also higher in the AKI group. Baseline serum creatinine was lower among infants who subsequently met the AKI criterion (21.3 vs. 24.4 μmol/L; *P* < 0.001), a difference relevant to interpretation of a creatinine-defined endpoint. Postoperative mechanical ventilation was longer and PD was more frequent in the AKI group.

**Table 1:** Baseline characteristics of study population.

Variable	Overall (*n* = 230)	No AKI (*n* = 173)	AKI (*n* = 57)	*p*-value
Demographics				
Age (months)	6 (4–9)	7 (4–10)	5 (3–7)	0.001
Weight (kg)	6.3 (5.2–7.4)	6.4 (5.4–7.7)	6.2 (4.7–7.0)	0.075
Height (cm)	64 (60–69.8)	65 (60–70)	62.5 (59–67)	0.018
BMI (kg/m²)	15.1 (13.8–16.2)	15.1 (13.8–16.2)	15.0 (13.9–16.0)	0.625
Preoperative laboratory				
Creatinine (μmol/L)	23.8 (20.5–26.8)	24.4 (21.2–26.9)	21.3 (18–25.3)	<0.001
Albumin (g/L)	41.2 (38.9–43.5)	41.3 (39–43.6)	41 (37.7–43.1)	0.357
Hemoglobin (g/L)	120 (109.2–128)	121 (110–128)	118 (108–129)	0.449
Platelet (×10^9^/L)	338 (267.5–410.5)	338 (262–415)	340 (280–398)	0.758
Surgical characteristics				
RACHS-1 score	2 (2–2)	2 (2–2)	2 (2–3)	0.034
CPB time (min)	93 (73–124)	91 (72–120)	105 (79–162)	0.038
Cross-clamp time (min)	55 (38–80)	52 (36.8–76)	63 (42–106)	0.035
Lowest temperature ( °C)	31.4 (30.4–32.1)	31.6 (30.6–32.1)	31.1 (29.5–32)	0.060
Blood loss (mL)	30 (20–50)	30 (20–50)	30 (20–50)	0.769
COP measurements				
Post-CPB COP (mmHg)	15.7 (14.2–17.4)	15.5 (14–17.1)	16.3 (15.1–18)	0.038
Prime COP (mmHg)	15.8 (13.7–18.9)	16 (13.8–19.5)	15 (13.5–17.8)	0.111
Pre-CPB COP (mmHg)	12.9 (12–14.4)	12.9 (12–14.4)	13.5 (12.3–14.5)	0.143
Intra-CPB COP (mmHg)	12.5 (11.8–13.8)	12.5 (12–13.8)	12.6 (11.5–13.7)	0.785
Perioperative management				
Fluid balance (mL/kg)	3.4 (–6.9–11.5)	4.3 (–5.7–11.5)	2.3 (–12.5–10.8)	0.243
Albumin dose (g/kg)	1 (0–1.8)	0.9 (0–1.7)	1.2 (0–2.4)	0.268
Total ultrafiltration (mL)	355 (300–460)	350 (300–450)	400 (300–500)	0.558
Postoperative outcomes				
Mechanical ventilation (h)	21 (8–41.6)	17 (7.9–26.8)	24 (15.5–70.7)	<0.001
Categorical variables				
Sex				0.646
Male	117 (50.9%)	86 (49.7%)	31 (54.4%)	
Female	113 (49.1%)	87 (50.3%)	26 (45.6%)	
Cyanotic status				0.735
Acyanotic	218 (94.8%)	163 (94.2%)	55 (96.5%)	
Cyanotic	12 (5.2%)	10 (5.8%)	2 (3.5%)	
Circulatory arrest				0.398
No	217 (94.3%)	165 (95.4%)	52 (91.2%)	
Yes	13 (5.7%)	8 (4.6%)	5 (8.8%)	
Death				0.061
No	228 (99.1%)	173 (100%)	55 (96.5%)	
Yes	2 (0.9%)	0 (0%)	2 (3.5%)	
Peritoneal dialysis				0.003
No	219/229 (95.6%)	169/172 (98.3%)	50/57 (87.7%)	
Yes	10/229 (4.4%)	3/172 (1.7%)	7/57 (12.3%)	

Data are presented as median (interquartile range) for continuous variables and *n* (%) for categorical variables. *P*-values were calculated using the Mann–Whitney U test for continuous variables and the *χ*^2^ test or Fisher's exact test for categorical variables, as appropriate. Postoperative PD status was available for 229 patients; one infant without AKI had missing PD status.

AKI, acute kidney injury; CPB, cardiopulmonary bypass; COP, colloid osmotic pressure.

Regarding ultrafiltration, 222 infants (96.5%) received both CUF and MUF. Median CUF volume among CUF recipients was 27.4 mL/kg (IQR, 19.5–38.5), and median MUF volume among MUF recipients was 20.8 mL/kg (IQR, 15.6–27.0) (Supplementary Table S1). Among the 57 infants with creatinine-defined AKI, 41 (71.9%) met the creatinine thresholds corresponding to Stage 1, 13 (22.8%) to Stage 2, and 3 (5.3%) to Stage 3. Postoperative PD was documented in 10 of 229 infants (4.4%), including 7 of 57 infants with AKI (Supplementary Table S2).

Cyanotic and acyanotic characteristics are shown in Supplementary Table S3. Only 12 infants (5.2%) had cyanotic disease. As expected, cyanotic infants had higher preoperative hemoglobin and pre-CPB hematocrit, but their pre-CPB COP was lower (11.6 vs. 13.1 mmHg; *P* = 0.008), while post-CPB COP was similar between groups (15.9 vs. 15.7 mmHg; *P* = 0.925). The cyanotic subgroup was too small for a reliable separate multivariable analysis. Weight-normalized blood-product exposure is also reported in Supplementary Table S3.

### 3.2 Association between post-CPB COP and AKI

Univariable analyses are summarized in Supplementary Table S4. In the prespecified multivariable model, higher post-CPB COP remained associated with AKI after adjustment for age, sex, body weight, RACHS-1 score, CPB duration, circulatory arrest, intraoperative fluid balance, and albumin dose. Each 1 mmHg higher post-CPB COP was associated with 16% higher odds of AKI (adjusted OR, 1.16; 95% CI, 1.04–1.28; *P* = 0.004; Supplementary Table S5). Younger age and longer CPB duration were also associated with AKI. The inverse association observed for recorded intraoperative fluid balance should be interpreted cautiously because this measure reflects both clinical management and underlying hemodynamic state.

### 3.3 Dose-response pattern and age-related heterogeneity

Restricted cubic spline analysis showed an approximately linear association between post-CPB COP and AKI, with no clear evidence of nonlinearity (*P* for nonlinearity = 0.114; Figure 2). COP was therefore retained as a continuous exposure in subsequent regression analyses.

**Figure 2:**
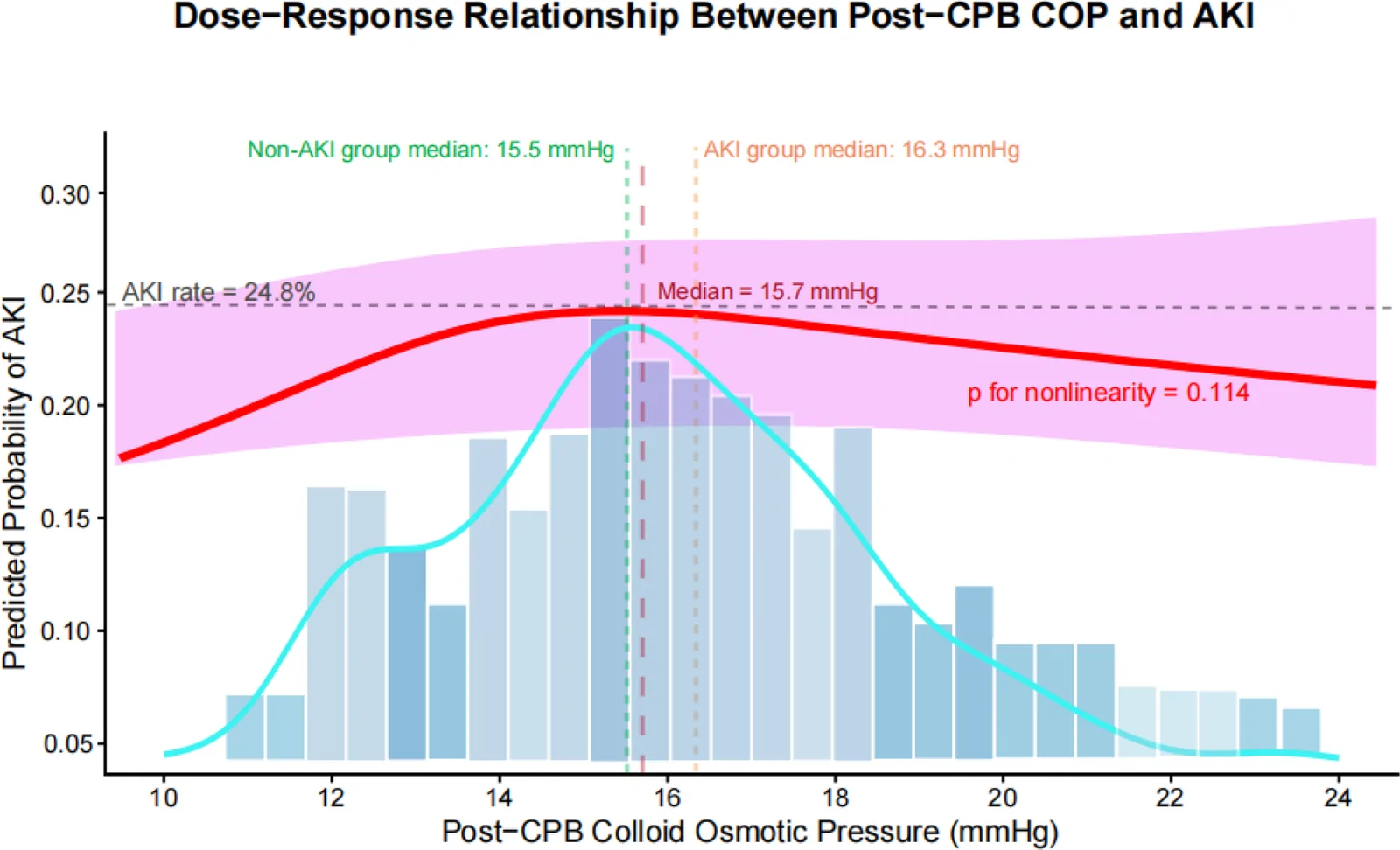
Dose-response association between post-CPB colloid osmotic pressure and postoperative AKI. The solid curve shows the adjusted predicted probability of AKI across post-CPB COP values, with the shaded band representing the 95% confidence interval. The horizontal dashed line indicates the overall AKI incidence and the vertical dotted line the median post-CPB COP. The model was adjusted for age, sex, body weight, RACHS-1 score, CPB duration, circulatory arrest, intraoperative fluid balance, and albumin dose. The test for nonlinearity was not significant (*P* = 0.114).

To explore whether the COP-AKI relationship varied across infancy, we fitted a GAM with a tensor-product smooth of COP and age. The joint smooth was statistically significant (*P* = 0.022; Figure 3; Supplementary Tables S6A–C), suggesting possible age-related heterogeneity in the shape of the association. Predicted risks and exploratory age-stratified logistic estimates are shown in Supplementary Table S7. The largest subgroup estimate was observed among infants aged 10–12 months (OR per 1 mmHg higher COP, 1.79; 95% CI, 1.09–3.64; *P* = 0.048), but the limited number of events and the borderline *P* value warrant cautious, hypothesis-generating interpretation. The age-COP prediction heatmap is provided in Supplementary Figure S3.

**Figure 3:**
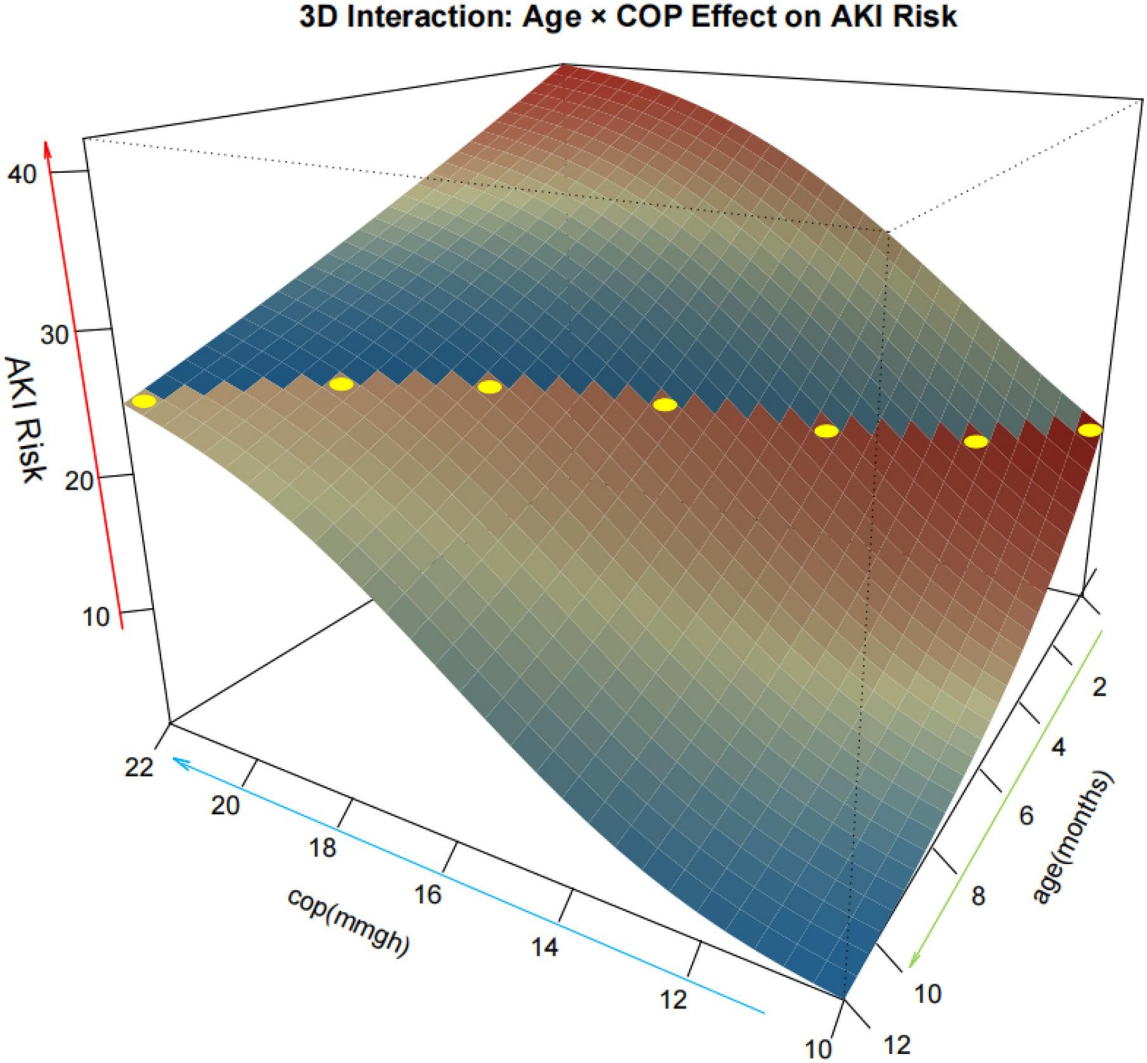
Joint age-COP pattern in model-predicted AKI risk. The heatmap shows model-predicted AKI risk across observed combinations of age and post-CPB COP from the generalized additive model adjusted for sex, body weight, RACHS-1 score, CPB duration, circulatory arrest, intraoperative fluid balance, and albumin dose. Darker shading denotes higher predicted risk. Marginal rug marks indicate the observed distributions of age and post-CPB COP; detailed counts for age-COP regions are provided in Supplementary Table S18.

### 3.4 Peri-CPB COP dynamics and time-specific associations

COP distributions were similar between the AKI and no-AKI groups at the pre-CPB and intra-CPB time points, with greater separation after CPB (Figure 4). In models using the same covariate framework as the primary analysis, pre-CPB COP (OR, 1.08; 95% CI, 0.95–1.23; *P* = 0.241) and intra-CPB COP (OR, 1.06; 95% CI, 0.89–1.27; *P* = 0.517) were not clearly associated with AKI, whereas the strongest time-specific association was observed for post-CPB COP (OR, 1.16; 95% CI, 1.04–1.28; *P* = 0.004). Neither the pre-to-post nor intra-to-post change in COP showed a clear association (Supplementary Table S8). These findings indicate that the AKI-related information carried by COP was concentrated at the end of CPB rather than in the magnitude of change alone.

**Figure 4:**
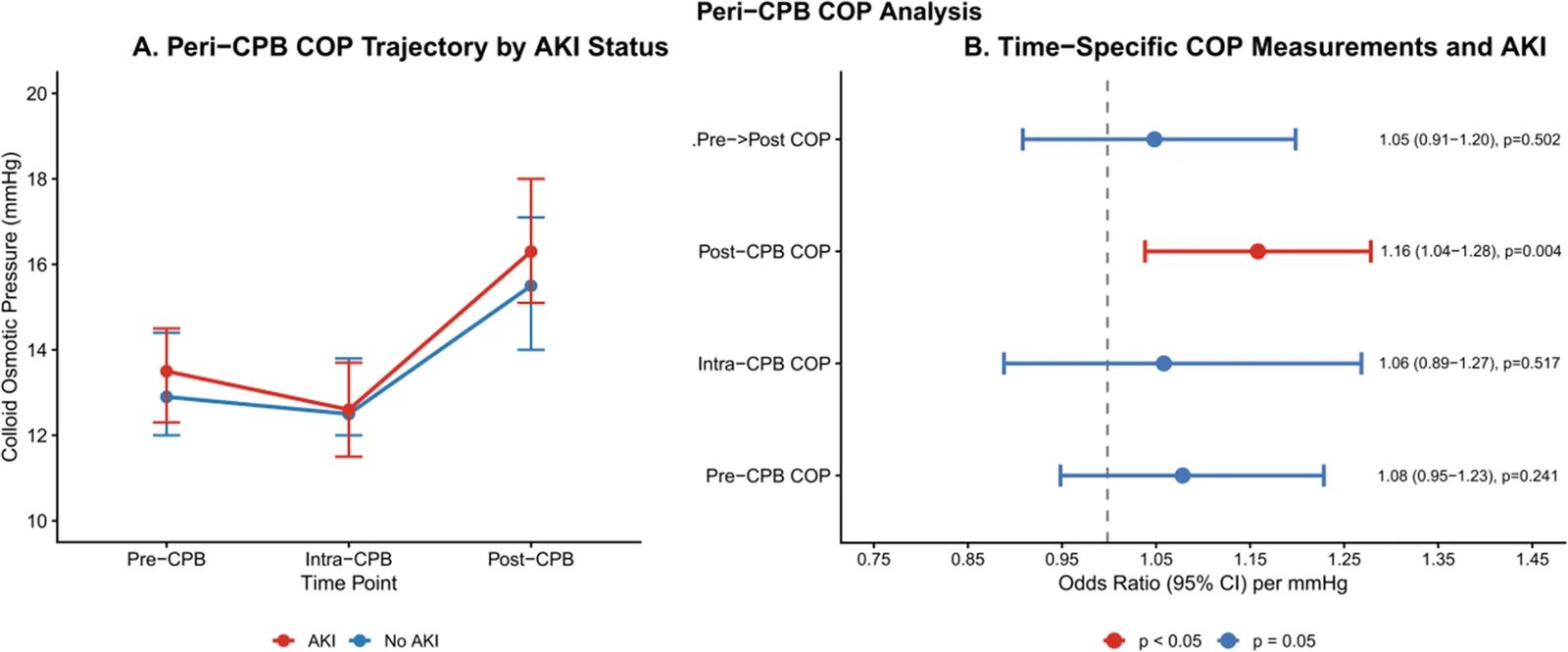
Peri-CPB COP dynamics and time-specific associations with AKI. **(A)** Peri-CPB COP trajectory by AKI status. COP values at pre-CPB, intra-CPB, and post-CPB are presented as median and interquartile range for infants with and without creatinine-defined AKI. **(B)** Time-specific peri-CPB COP measurements and AKI. Adjusted ORs and 95% CIs are shown for pre-CPB, intra-CPB, post-CPB COP, and changes from pre-to-post and intra-to-post. Each time-specific exposure was modeled separately using the prespecified adjustment framework.

### 3.5 Exploratory interaction analyses

No statistically significant multiplicative interaction was observed between post-CPB COP and intraoperative fluid balance (interaction OR, 1.002; 95% CI, 0.996–1.009) or albumin dose (interaction OR, 0.963; 95% CI, 0.876–1.059 (Figure 5); Supplementary Table S9). A regression-based mediation framework was also explored, but intraoperative fluid balance occurred before the post-CPB COP measurement. Accordingly, the resulting indirect/direct-effect decomposition (Supplementary Table S10 and Figure S4) is presented only as an exploratory statistical analysis and should not be interpreted as evidence of causal mediation.

**Figure 5:**
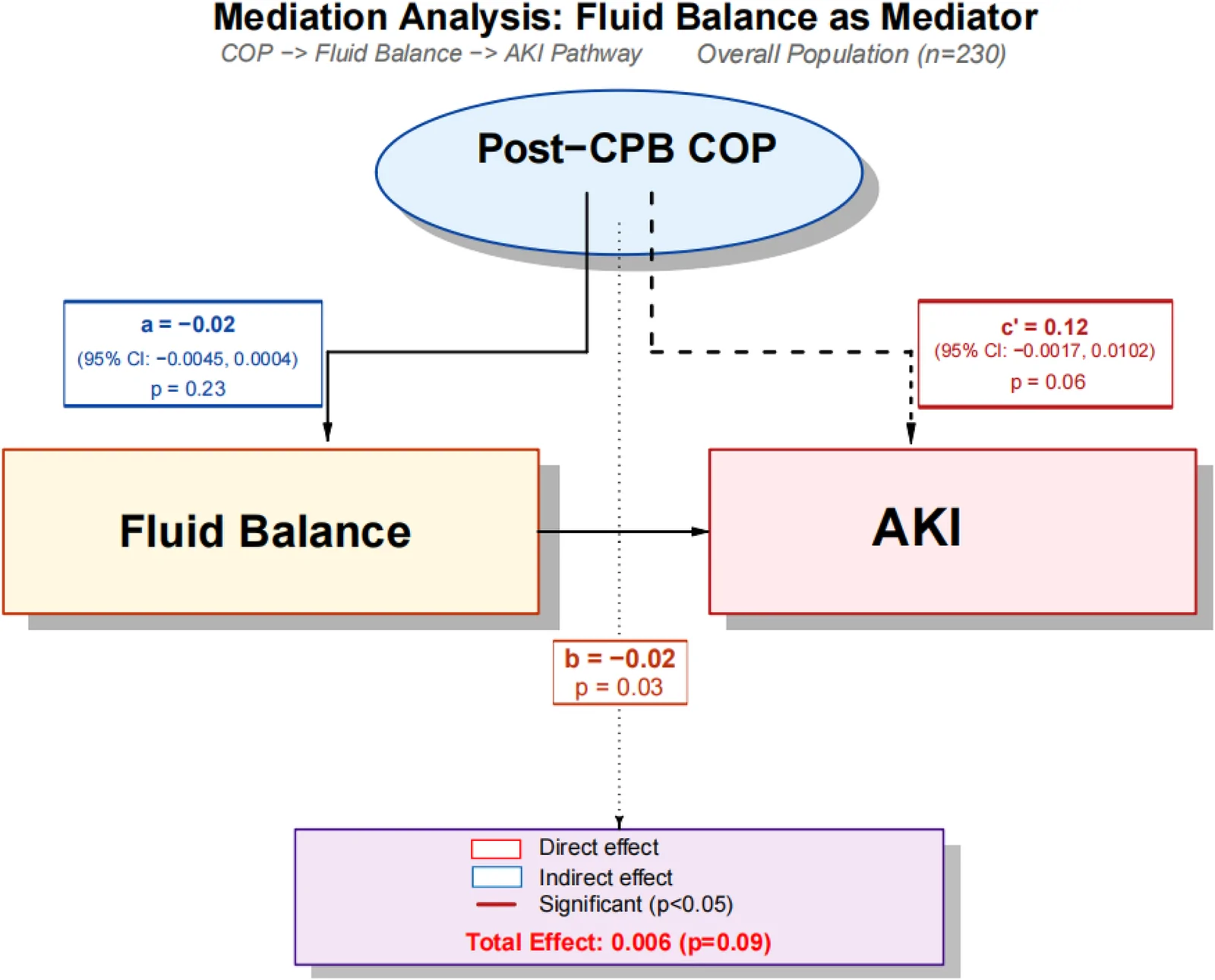
Exploratory statistical path diagram relating intraoperative fluid balance, post-CPB COP, and AKI. Figure caption: This diagram summarizes a regression-based statistical decomposition among intraoperative fluid balance, post-CPB COP, and postoperative AKI. Because intraoperative fluid balance preceded the post-CPB COP measurement, arrow direction should not be interpreted as establishing temporal causal mediation. The overall indirect-effect estimate was close to zero (−0.0008; 95% CI, −0.0045 to 0.0004), and the analysis is presented for descriptive sensitivity purposes only.

### 3.6 Predictive performance and incremental value of COP

COP alone had limited discrimination for AKI (AUC, 0.592). Adding COP to the clinical model increased the AUC from 0.701 to 0.723, but the increment was not statistically significant (DeLong *P* = 0.324; Supplementary Table S11 and Figure S5). NRI was 0.070 (95% CI, −0.062 to 0.196), with the confidence interval crossing zero (Supplementary Table S12). Calibration of the full model was acceptable by the Hosmer-Lemeshow test (*P* = 0.638; Supplementary Figure S6).

Decision curve analysis showed only marginal overall improvement after adding COP, limited to a narrow range of threshold probabilities (Supplementary Figure S7). In exploratory age-stratified analyses, the largest point estimate for incremental net benefit was observed in the 10–12-month group (+0.0417), whereas estimates were minimal in younger strata (Supplementary Figure S8 and Table S13). Given the small subgroup sizes and absence of external validation, these findings should not be interpreted as evidence of established age-specific clinical utility.

### 3.7 Sensitivity analyses

Subgroup and exclusion analysis: After excluding neonates (<1 month), deceased cases, patients with preoperative abnormal renal function (preoperative Cr > 70 µmol/L), and those with extreme COP values (<10 or >22 mmHg), the estimated effect of COP remained consistent with the direction observed in the main analysis. No reversal in direction was observed in either the albumin or fluid balance subgroups (Supplementary Figure S9, and Table S14); regarding ultrafiltration and peri-CPB management factors: no clear linear relationship was observed between CPB duration and total ultrafiltration volume (regression coefficient: 0.025 mL/kg/min, 95% CI: −0.022 to 0.073, *P* = 0.294), nor between total ultrafiltration volume and post-CPB COP (regression coefficient: 0.010 mmHg/mL/kg, 95% CI: −0.007 to 0.028, *P* = 0.252). When total ultrafiltration volume was added to the main model, the OR for COP decreased slightly from 1.16 (95% CI: 1.04–1.28) to 1.13 (95% CI: 0.99–1.29). When further adjusting for preoperative albumin levels, excluding patients with hypoalbuminemia (<35 g/L), and analyzing only non-cyanotic infants, both the direction and magnitude of the COP effect remained similar; however, the corresponding confidence intervals widened, approaching or even crossing the null value (Supplementary Table 15). Model specification and collinearity assessment: The aortic cross-clamping duration was highly correlated with the CPB duration (r = 0.927, VIFs of 9.04 and 8.34, respectively), indicating severe multicollinearity; therefore, the main model selected the CPB duration as the representative variable for surgical complexity. When the cross-clamping duration was used as an alternative to the CPB duration, the estimated effect of COP remained essentially unchanged (OR 1.23,95% CI 1.06–1.43, *P* = 0.005) (Supplementary Table S16). Across different model specifications (with or without adjustment for intraoperative fluid balance, albumin levels, and ultrafiltration volume), the direction and magnitude of the COP effect remained consistent (Supplementary Table S17), supporting the robustness of the primary findings; IPTW and quartile analysis: The IPTW analysis based on a 16 mmHg dichotomized COP classification showed a consistent direction but imprecise estimates (OR: 1.59,95% CI: 0.85–2.95, *P* = 0.14). When stratified by COP quartiles, the incidence of AKI showed a graded increase (16.9%, 21.1%, 30.0%, and 31.5% for Q1 to Q4), with a Cochran–Armitage trend test yielding *P* = 0.039 (Supplementary Figures S10–S11). For unmeasured confounding factors: the E-value corresponding to the point estimate was 1.59, while the E-value corresponding to the lower limit of the 95% confidence interval was 1.24. This indicates that an unmeasured confounding factor with an association strength corresponding to a hazard ratio of 1.59 for both COP and AKI could fully account for the observed effect. Therefore, moderate unmeasured confounding may weaken the conclusions of this study.

## 4 Discussion

### 4.1 Principal findings

In this single-center cohort of infants undergoing cardiac surgery with CPB, higher post-CPB COP was associated with creatinine-defined postoperative AKI after covariate adjustment. The dose-response pattern was approximately linear. Time-specific analyses added important context: pre-CPB and intra-CPB COP were not clearly associated with AKI, whereas the strongest signal was concentrated at the end of CPB. Changes in COP between time points were not clearly associated with AKI. These observations suggest that the relevant information may lie less in the absolute rise in COP than in the physiologic state present at CPB separation—a state shaped by hemodilution, albumin exposure, ultrafiltration, hemoconcentration, and perfusion. COP therefore appears more useful as a time-specific perioperative risk marker than as an isolated laboratory value or established therapeutic target. Its incremental predictive value beyond conventional clinical variables was limited.

The age-related findings require a similarly restrained interpretation. The joint age-COP GAM surface suggested heterogeneity across infancy, and the largest exploratory subgroup estimate occurred in infants aged 10–12 months. However, subgroup event counts were limited, confidence intervals were wide, and statistical significance in one age band does not establish absence of association in the others. These analyses should therefore be considered hypothesis-generating rather than evidence for a discrete age-specific susceptibility window.

### 4.2 Developmental physiology and age-dependent susceptibility

Renal maturation during infancy is a dynamic rather than a uniform process. Quantitative analyses of measured glomerular filtration rate have shown a rapid postnatal increase in renal filtration capacity, with maturation continuing throughout infancy (27). Tubular development follows a related but not identical trajectory; tubular growth, transport capacity, and responses to increasing postnatal filtrate flow continue to evolve well beyond the neonatal period (28). Prematurity adds another layer of developmental heterogeneity because interruption of late gestational nephrogenesis may reduce nephron endowment and alter subsequent renal functional reserve (29). These developmental differences provide a biologically plausible basis for examining whether the physiologic meaning of a peri-CPB exposure differs across infancy.

The larger COP estimate observed among infants aged 10–12 months should nevertheless be interpreted cautiously. Younger infants have a greater background burden of developmental and perioperative susceptibility, whereas in later infancy specific perioperative exposures may become more distinguishable from this background risk. At the same time, increasing glomerular filtration and continued tubular maturation alter the balance between filtered load, active tubular transport, and oxygen demand (27, 28). These considerations make an age-related pattern physiologically plausible, but they do not establish late infancy as a specific period of susceptibility to elevated COP. Given the limited number of AKI events within individual age strata, the 10–12-month finding remains hypothesis-generating.

### 4.3 Potential mechanisms linking COP and AKI

Low COP has traditionally received attention because reduced oncotic pressure may facilitate interstitial edema and alter transvascular fluid exchange. However, a higher post-CPB COP should not automatically be interpreted as physiologically favorable. In the present setting, higher COP may instead identify a broader hemoconcentration phenotype arising from ultrafiltration, colloid exposure, changes in plasma protein concentration, and other components of CPB management. Importantly, COP and blood viscosity are distinct physiologic variables and should not be treated as interchangeable.

Nevertheless, pediatric evidence supports a potential link between hemorheology and renal stress during CPB. In infants weighing <10 kg, Dittrich et al. demonstrated that changes in blood viscosity during hypothermic CPB were associated with markers of glomerular and tubular injury, suggesting that blood rheology may influence renal tolerance during extracorporeal circulation (30).This observation does not establish that elevated COP causes increased viscosity, but it supports the possibility that COP and rheologic changes may coexist within the same hemoconcentration phenotype.

Renal microvascular vulnerability provides a second potential link. The renal medulla receives a relatively small proportion of total renal blood flow despite substantial tubular oxygen requirements, making this region particularly susceptible to disturbances in oxygen delivery (31). Experimental ischemia–reperfusion studies further demonstrate persistent postischemic capillary constriction and medullary no-reflow mediated by pericytes, providing a mechanistic basis by which apparently restored systemic circulation may coexist with impaired renal microvascular perfusion (32).

In our cohort, recorded intraoperative fluid balance did not account for the observed COP–AKI association. This does not imply a direct nephrotoxic effect of COP. Rather, post-CPB COP may integrate information from hemodilution, ultrafiltration, albumin exposure, hemoconcentration, and unmeasured perfusion characteristics. The absence of continuous hematocrit, oxygen delivery, perfusion pressure, lactate, venous oxygen saturation, and low-flow duration therefore remains particularly relevant because these variables may represent core components of the physiologic state captured indirectly by post-CPB COP.

### 4.4 Comparison with previous studies

Pediatric cardiac surgery-associated AKI has been described across a broad range of congenital heart surgery populations. In the multicenter NEPHRON cohort, AKI occurred frequently after neonatal cardiac surgery and showed substantial interinstitutional variation, emphasizing the heterogeneity of the postoperative renal phenotype (33). Fluid status is also closely linked to postoperative renal outcomes; Hassinger et al. showed that early fluid overload can precede AKI and is associated with greater morbidity after pediatric cardiac surgery (34).

More recent work has increasingly emphasized the importance of perfusion and oxygen delivery rather than isolated static variables. In infants undergoing CPB, lower nadir indexed oxygen delivery has been associated with postoperative AKI (35), while renal or peripheral tissue oxygenation assessed using near-infrared spectroscopy has also shown associations with subsequent kidney injury (36, 37).

Against this background, our study contributes a different but complementary observation. Rather than evaluating oxygen delivery or fluid overload directly, we examined serial, directly measured peri-CPB COP. The fact that the association was most apparent for the post-CPB measurement, while earlier COP values and changes between time points were not clearly associated with AKI, suggests that the physiologic state present at the end of bypass may be more informative than a universal COP threshold.

### 4.5 Clinical implications

The present findings do not support immediate adoption of a numerical COP treatment target. Instead, post-CPB COP may be most useful as a signal prompting broader assessment of the physiologic context in which the value was generated. In an infant with a relatively high post-CPB COP, clinicians may reasonably review the degree of hemoconcentration, ultrafiltration exposure, hematocrit, albumin administration, perfusion pressure, and oxygen delivery rather than responding to COP in isolation. This interpretation is consistent with pediatric studies showing that renal risk during CPB is closely linked to oxygen-delivery and tissue-oxygenation profiles. Importantly, our RCS analysis did not identify a reproducible COP threshold, and the exploratory 16-mmHg analysis should not be interpreted as defining a treatment target. Prospective intervention studies would be required before any COP-guided strategy or age-specific target range could be recommended.

### 4.6 Strengths and limitations

Several features strengthen this study. The cohort was restricted to infants undergoing CPB, COP was measured directly at prespecified peri-CPB time points rather than inferred from albumin or fluid balance, and serial peri-CPB analyses placed the primary exposure in temporal context. Prespecified regression, flexible modeling, and multiple sensitivity analyses were used to examine consistency rather than relying on a single model.

The study also has important limitations. First, its retrospective single-center design leaves potential for selection bias, measurement error, and residual confounding. Second, AKI was defined using serum creatinine within a prespecified 48-hour window because sufficiently granular postoperative urine-output data were unavailable; oliguric AKI may therefore have been missed. Baseline creatinine was lower in infants who subsequently met the AKI criterion, which may partly reflect age and muscle mass but also affects the mathematical probability of satisfying a creatinine-based endpoint. Alternative models including baseline creatinine produced directionally consistent COP estimates, but classification bias cannot be fully excluded. Third, only 57 AKI events occurred, and age-stratified analyses were correspondingly imprecise. Fourth, the dataset included pre-, intra-, and post-CPB COP but not serial ICU COP, so persistent elevation, recovery, or secondary postoperative changes could not be characterized. A true untreated preoperative COP was also unavailable.

Fifth, early postoperative fluid balance, hourly urine output, diuretic use, and resuscitation were incomplete. Intraoperative fluid balance itself was derived from routine clinical records and did not capture all unrecovered losses, laboratory sampling, or insensible losses. Sixth, only 12 infants had cyanotic disease, limiting adjustment for chronic hypoxemia and disease phenotype.

Finally, key perfusion and hemodynamic variables—including continuous or nadir hematocrit, indexed oxygen delivery, intra-CPB mean arterial pressure, renal perfusion pressure, vasoactive exposure, lactate trajectory, venous oxygen saturation, low-flow duration, and postoperative low-cardiac-output syndrome—could not be consistently reconstructed from the available records. This gap is particularly relevant for blood pressure, which directly influences renal oxygen delivery and glomerular filtration. Intraoperative hypotension may compound the effects of hemoconcentration and elevated COP on renal medullary oxygenation, and we could not determine whether the COP-AKI association was modified by intraoperative hemodynamic status. These missing variables are central to the physiologic interpretation of COP and may confound or mediate its association with AKI.

### 4.7 Future research directions

Future studies should move beyond isolated COP measurements and reconstruct, prospectively, the temporal sequence that links perfusion management to kidney injury. Ideally, serial COP values would be interpreted alongside contemporaneous hematocrit, plasma protein concentration, ultrafiltration intensity, indexed oxygen delivery, perfusion pressure, vasoactive support, and postoperative hemodynamics. Repeated assessment of renal tissue oxygenation may help establish whether changes in COP precede renal oxygenation abnormalities, and postoperative renal Doppler indices could provide complementary information on renal vascular resistance and perfusion (38). Such studies would allow direct testing of the proposed sequence: peri-CPB management → COP/hemoconcentration phenotype → altered renal perfusion or oxygenation → tubular injury → clinical AKI. Interventional trials designed to manipulate COP should therefore be deferred until this temporal relationship has been established. Beyond the perioperative window, long-term follow-up of renal function—including eGFR trajectory, proteinuria, and hypertension—will be needed to determine whether the COP-AKI association observed in infancy translates into lasting renal sequelae.

## 5 Conclusion

Higher post-CPB COP was associated with creatinine-defined AKI after infant cardiac surgery, and the clearest risk information was observed immediately after CPB. Exploratory analyses suggested that the association may vary across infancy, but age-stratified findings require cautious interpretation. COP provided limited incremental predictive value and no validated treatment threshold was identified. At present, post-CPB COP is best viewed as a time-specific physiologic risk marker to be interpreted alongside hemoconcentration, colloid exposure, and perfusion context rather than as an independent prediction tool or therapeutic target.

## Data Availability

The raw data supporting the conclusions of this article will be made available by the authors, without undue reservation.
