# Supplementary material for: Post-cardiopulmonary bypass colloid osmotic pressure and creatinine-defined acute kidney injury in infants undergoing cardiac surgery: an age-modified association
Source: Front Pediatr. 2026 Oct 14;14:1929104. doi: 10.3389/fped.2026.1929104 (PMC13713631; doi:10.3389/fped.2026.1929104)
Supplement: Supplementary file 1 [file Supplementaryfile1.docx]

Supplemental Digital Content

**Supplemental Digital Content — Table of Contents**

| **Item** | **Page** |
| --- | --- |
| Supplementary Table 1. Ultrafiltration Strategies and Weight-Normalized Ultrafiltration Volumes | 2 |
| Supplementary Table 2. Creatinine-Defined AKI Severity and Postoperative Peritoneal Dialysis | 3 |
| Supplementary Table 3. Cyanosis-Related Characteristics and Intraoperative Blood-Product Exposure | 3 |
| Supplementary Table 4. Univariable Logistic Regression for AKI | 4 |
| Supplementary Table 5. Multivariable Logistic Regression for AKI | 5 |
| Supplementary Table 6A. Parametric Terms from GAM | 5 |
| Supplementary Table 6B. Smooth Terms from GAM | 6 |
| Supplementary Table 6C. Model Fit Statistics | 6 |
| Supplementary Table 7. GAM-Predicted AKI Risk Across Age–COP Combinations and Exploratory Age-Stratified Logistic Associations | 7 |
| Supplementary Table 8. Time-Specific Peri-CPB COP Measurements and AKI | 7 |
| Supplementary Table 9. Interaction Tests: COP × Fluid Balance and COP × Albumin | 8 |
| Supplementary Table 10. Mediation Analysis: Fluid Balance and Albumin as Mediators | 9 |
| Supplementary Table 11. Model Performance for AKI Prediction | 10 |
| Supplementary Table 12. Net Reclassification Improvement (NRI) After Adding COP to Clinical Model | 11 |
| Supplementary Table 13. Age-Dependent Clinical Utility of COP: Decision Curve Analysis Results | 12 |
| Supplementary Table 14. Subgroup Analysis | 13 |
| Supplementary Table 15. Sensitivity Analysis of Ultrafiltration and Peri-CPB Management Factors | 14 |
| Supplementary Table 16. Collinearity Assessment and Sensitivity Analysis for Cross-Clamp Time | 14 |
| Supplementary Table 17. Model Specification Sensitivity Analysis for the COP–AKI Association | 15 |
| Supplementary Table 18. Sample Density by Age and COP Region | 16 |
| Supplementary Figure 1. Directed Acyclic Graph of the Temporal and Causal Framework for Post-CPB COP and AKI | 17 |
| Supplementary Figure 2. Age-Specific Dose-Response Curve of Postoperative COP and AKI Risk | 18 |
| Supplementary Figure 3. Age–COP Joint Effect Heatmap | 20 |
| Supplementary Figure 4. Age-Stratified Mediating Path Diagram: Fluid Balance in Infants ≤6 Months vs >6 Months | 20 |
| Supplementary Figure 5. ROC Curves for Three Prediction Models | 21 |
| Supplementary Figure 6. Calibration Curve for the Full Model | 22 |
| Supplementary Figure 7. Population-Wide Decision Curve | 23 |
| Supplementary Figure 8. Age-Stratified Decision Curve | 24 |
| Supplementary Figure 9. Forest Plot of Sensitivity Analysis | 24 |
| Supplementary Figure 10. Comparison of Standardized Mean Differences Before and After IPTW Weighting | 25 |
| Supplementary Figure 11. Postoperative COP Quartiles and AKI Risk | 27 |

**Supplementary Table 1. Ultrafiltration Strategies and Weight-Normalized Ultrafiltration Volumes**

Panel A. Ultrafiltration strategy distribution

| UF strategy | n (%) | Total UF volume, mL/kg |
| --- | --- | --- |
| CUF + MUF | 222 (96.5%) | 40.0 (33.3–49.5) |
| CUF only | 3 (1.3%) | 35.7 (28.3–46.2)* |
| MUF only | 4 (1.7%) | 27.5 (22.1–36.8)* |
| Neither | 1 (0.4%) | 0 |

Panel B. Technique-specific ultrafiltration volumes

| Technique | Patients receiving technique, n (%) | Volume, mL/kg |
| --- | --- | --- |
| CUF | 225 (97.8%) | 27.4 (19.5–38.5) |
| MUF | 226 (98.3%) | 20.8 (15.6–27.0) |

*For subgroups with n ≤4, data are presented as median (range); otherwise values are median (IQR). CUF, conventional ultrafiltration; MUF, modified ultrafiltration; UF, ultrafiltration.

**Supplementary Table 2. Creatinine-Defined AKI Severity and Postoperative Peritoneal Dialysis**

Panel A. Creatinine-defined AKI severity in the entire cohort

| AKI category | n | % of total cohort | % among AKI |
| --- | --- | --- | --- |
| No creatinine-defined AKI | 173 | 75.2% | — |
| Stage 1 | 41 | 17.8% | 71.9% |
| Stage 2 | 13 | 5.7% | 22.8% |
| Stage 3 | 3 | 1.3% | 5.3% |

Panel B. Postoperative peritoneal dialysis (available-case analysis)

| PD status | Overall (n=229) | No AKI (n=172) | AKI (n=57) |
| --- | --- | --- | --- |
| No PD | 219 (95.6%) | 169 (98.3%) | 50 (87.7%) |
| PD | 10 (4.4%) | 3 (1.7%) | 7 (12.3%) |

AKI severity categories are based on the serum creatinine component of KDIGO because duration-based urine-output criteria were not reliably available. PD was reported separately from the prespecified creatinine-defined AKI classification. PD status was missing for one infant without AKI.

**Supplementary Table 3. Cyanosis-Related Characteristics and Intraoperative Blood-Product Exposure**

Panel A. Cyanotic versus acyanotic characteristics

| Variable | Acyanotic (n=218) | Cyanotic (n=12) | P value |
| --- | --- | --- | --- |
| Preoperative hemoglobin, g/L | 119 (109–127) | 156 (127–183) | <0.001 |
| Pre-CPB hematocrit, % | 33 (30.2–35) | 39 (36–49) | <0.001 |
| Pre-CPB COP, mmHg | 13.1 (12.0–14.4) | 11.6 (10.9–13.0) | 0.008 |
| Post-CPB COP, median, mmHg | 15.7 | 15.9 | 0.925 |
| Creatinine-defined AKI | 55/218 (25.2%) | 2/12 (16.7%) | 0.735* |

*Fisher exact test. Continuous variables were compared using the Wilcoxon rank-sum test. The cyanotic subgroup was small; these comparisons are descriptive.

Panel B. Intraoperative blood-product exposure

| Blood-product exposure | Patients exposed, n (%) | Volume, mL/kg |
| --- | --- | --- |
| RBC in CPB prime | 192 (83.5%) | 4.8 (3.1–8.2) |
| Additional intraoperative PRBC | 3 (1.3%) | 6.8, 11.4, 11.5† |
| Intraoperative FFP | 23 (10.0%) | 6.2 (3.8–11.0) |

†Individual weight-normalized values are shown because only three infants received additional intraoperative PRBC. Blood-product volumes were normalized to body weight. RBC in CPB prime was converted from recorded units using the institution-specific nominal volume used in the source dataset.

Supplementary Table 4. Univariable Logistic Regression for AKI

| **Variable** | **OR** | **95% CI** | **P_value** |
| --- | --- | --- | --- |
| pre_cr | 0.87 | 0.81-0.93 | <0.001 |
| vent_time | 1.01 | 1.011.02 | <0.001 |
| age | 0.85 | 0.77-0.93 | <0.001 |
| cpb_time | 1.01 | 1-1.02 | 0.0026 |
| cross_time | 1.01 | 1-1.02 | 0.0039 |
| temp_min | 0.84 | 0.73-0.96 | 0.012 |
| rachs | 1.68 | 1.04-2.71 | 0.0347 |
| weight | 0.83 | 0.69-1.01 | 0.0576 |
| cop_post | 1.16 | 1.06–1.26 | 0.002 |
| height | 0.97 | 0.94-1 | 0.0799 |
| blood_loss | 1.01 | 1-1.02 | 0.1097 |
| albumin | 1.21 | 0.93-1.56 | 0.1493 |
| fluid_balance | 0.99 | 0.97-1 | 0.1559 |
| pre_alb | 1.01 | 0.99-1.02 | 0.3071 |
| cop_pre | 1.06 | 0.94-1.2 | 0.3241 |
| cop_prime | 0.97 | 0.91-1.04 | 0.422 |
| pre_hgb | 0.99 | 0.97-1.01 | 0.4247 |
| total_uf | 1 | 1-1 | 0.7312 |
| pre_plt | 1 | 1-1 | 0.805 |
| cop_intra | 0.99 | 0.83-1.17 | 0.8873 |

**Table Note:** A single-factor logistic regression model was employed to evaluate the association

between each potential risk factor and postoperative acute kidney injury (AKI).

Supplementary Table 5. Multivariable Logistic Regression for AKI

| **Variable** | **OR** | **95% CI** | **p-value** |
| --- | --- | --- | --- |
| Post-CPB COP (per mmHg) | 1.16 | 1.04-1.28 | **0.004** |
| Age (per month) | 0.79 | 0.67-0.92 | **0.003** |
| Male sex | 1.24 | 0.63-2.48 | 0.538 |
| Weight (per kg) | 1.13 | 0.79-1.65 | 0.500 |
| RACHS-1 score | 1.66 | 0.90-3.04 | 0.101 |
| CPB time (per min) | 1.01 | 1.00-1.02 | **0.011** |
| Circulatory arrest | 0.56 | 0.12-2.27 | 0.434 |
| Fluid balance (per mL/kg) | 0.98 | 0.96-1.00 | **0.034** |
| Albumin dose (per g/kg) | 0.74 | 0.48-1.14 | 0.174 |

**Table note:** variables with P<0.10 in univariate analysis, known clinical risk

factors (even if P≥0.10 in univariate analysis), and avoidance of multicollinearity (variance

inflation factor VIF<5). The final included variables were: postoperative COP, age, sex, body

weight, RACHS-1 score, CPB duration, circulatory arrest, and fluid balance.Abbreviations: AKI,

acute kidney injury; CPB, cardiopulmonary bypass; COP, colloid osmotic pressure; CI, confidence interval.

Supplementary Table 6A. Parametric Terms from GAM

| **Component** | **Variable** | **OR** | **CI_lower** | **CI_upper** | **P_value** |
| --- | --- | --- | --- | --- | --- |
| Parametric terms | Fluid balance (per mL/kg) | 0.98 | 0.96 | 1.00 | **0.0264** |
| Parametric terms | Gender (male vs female) | 1.21 | 0.61 | 2.42 | 0.5837 |
| Parametric terms | Weight (per kg) | 1.13 | 0.78 | 1.64 | 0.5228 |
| Parametric terms | RACHS-1 score | 1.63 | 0.89 | 3.00 | 0.1134 |
| Parametric terms | CPB time (per min) | 1.01 | 1.00 | 1.02 | **0.0120** |
| Parametric terms | Circulatory arrest (yes vs no) | 0.61 | 0.15 | 2.57 | 0.5023 |
| Parametric terms | Albumin dose (per g/kg) | 0.78 | 0.51 | 1.21 | 0.2674 |

Table Note：This table presents the effects of covariates included as linear terms in the

generalized additive model (GAM). The model was adjusted for fluid balance, gender, weight,

RACHS-1 score, CPB time, circulatory arrest, and albumin dose. OR: odds ratio; CI: 95%

confidence interval. Fluid balance (P = 0.0264) and CPB time (P = 0.0120) demonstrated

independent linear associations with acute kidney injury (AKI), consistent with the multivariable

logistic regression results. No other covariates reached statistical significance.

Supplementary Table 3B. Smooth Terms from GAM

| **Component** | **Variable** | **EDF** | **Ref_df** | **Chi_sq** | **P_value** |
| --- | --- | --- | --- | --- | --- |
| Smooth terms | COP × age interaction | 3.921 | 4.520 | 12.270 | ***0.0217*** |

Table Note：The smooth term te(cop_post, age) represents the tensor product interaction between

COP and age, which was statistically significant (p = 0.0217), indicating that the effect of COP on AKI varies nonlinearly with age.Abbreviations: GAM, generalized additive model; COP, colloid osmotic pressure; CPB, cardiopulmonary bypass; EDF, effective degrees of freedom; REML, restricted maximum likelihood; AIC, Akaike information criterion.

Supplementary Table 3C. Model Fit Statistics

| **Component** | **Metric** | **Value** |
| --- | --- | --- |
| Model fit | Deviance explained | 12.8% |
| Model fit | REML score | 120.19 |
| Model fit | AIC | 249.72 |
| Model fit | n | 230 |

**Table Note:** To investigate the potential nonlinear relationship between postoperative COP and AKI as well as the moderating effect of age, we constructed a Generalized Additive Model (GAM) incorporating a tensor product smoothing term for COP and age. The results demonstrated statistically significant interaction between COP and age (EDF = 3.92, χ² = 12.27, P = 0.022) (Table 3), indicating that the impact of COP on AKI varies nonlinearly with age. The parameter estimates in the model (see Supplementary Table S3) were consistent with traditional logistic regression, further confirming the independent effects of fluid balance (OR 0.98,95% CI 0.96–1.00, P = 0.026) and CPB duration (OR 1.01,95% CI 1.00–1.02, P = 0.012).

Supplementary Table 7. GAM-Predicted AKI Risk Across Age–COP Combinations and Exploratory Age-Stratified Logistic Associations

| **Age_Group** | **10-12 mmHg** | **13-15 mmHg** | **16-18 mmHg** | **19-22 mmHg** | **P.value（GAM predicted risk）** | **Logistic Exploratory stratified OR per 1 mmHg increase in COP** | **P_value（Logistic ）** |
| --- | --- | --- | --- | --- | --- | --- | --- |
| 0-3 months | 26.1% (8.6-57.0) | 29.6% (13.5-53.0) | 33.6% (17.1-55.4) | 37.5% (16.3-64.9) | <0.001 | 1.06 (0.87-1.29) | 0.584 |
| 4-6 months | 22.5% (10.6-41.6) | 26.9% (17.6-38.9) | 32.3% (23.0-43.2) | 37.7% (22.1-56.4) | <0.001 | 1.08 (0.86-1.36) | 0.522 |
| 7-9 months | 10.2% (3.6-25.8) | 14.7% (8.1-25.1) | 21.3% (13.6-31.6) | 29.0% (14.3-50.0) | <0.001 | 1.01 (0.71-1.40) | 0.956 |
| 10-12 months | 2.5%  (0.4-14.5) | 4.8%  (1.5-14.2) | 9.5%  (3.8-21.8) | 17.2% (4.4-48.3) | <0.001 | 1.79 (1.09-3.64) | 0.048 |

**Table Note:** Values in the four COP columns represent model-predicted probabilities of postoperative acute kidney injury (AKI) derived from the generalized additive model (GAM) incorporating a tensor product smooth for postoperative colloid osmotic pressure (COP) and age. Predicted probabilities are presented as percentages with 95% confidence intervals. The GAM was adjusted for sex, body weight, RACHS-1 score, cardiopulmonary bypass duration, circulatory arrest, fluid balance, and albumin dose. The last two columns report exploratory age-stratified logistic regression results estimating the association between each 1 mmHg increase in postoperative COP and AKI within each age group. These stratified ORs and P values were derived from separate logistic regression models and should be interpreted as exploratory subgroup analyses rather than as formal tests of the GAM-predicted probabilities. Overall, the GAM suggested increasing predicted AKI risk with higher postoperative COP across age strata, whereas exploratory stratified logistic regression showed a statistically significant association only among infants aged 10–12 months. Given the limited number of events within age strata, these subgroup findings should be interpreted cautiously.

**Supplementary Table 8. Time-Specific Peri-CPB COP Measurements and AKI**

| COP exposure | Adjusted OR | 95% CI | P value |
| --- | --- | --- | --- |
| Pre-CPB COP | 1.08 | 0.95–1.23 | 0.241 |
| Intra-CPB COP | 1.06 | 0.89–1.27 | 0.517 |
| Post-CPB COP | 1.16 | 1.04–1.28 | 0.004 |
| ΔCOP, pre→post | 1.05 | 0.91–1.20 | 0.502 |
| ΔCOP, intra→post | 1.10 | 0.96–1.27 | 0.170 |

Each time-specific model was adjusted using the same prespecified covariate framework as the primary analysis. COP measurements from different time points were modeled separately rather than entered simultaneously. ΔCOP denotes the change in COP between the indicated peri-CPB time points.

Supplementary Table 9. Interaction Tests: COP × Fluid Balance and COP × Albumin

| **Interaction_Term** | **OR** | **95% CI** | **P_value** |
| --- | --- | --- | --- |
| COP × Fluid balance | 1.002 | 0.996–1.009 | 0.4616 |
| COP × Albumin | 0.963 | 0.876–1.059 | 0.4399 |

**Table Nate：**Models adjusted for age, gender, weight, RACHS-1, CPB time, circulatory arrest,

and either albumin (for COP×Fluid balance) or fluid balance (for COP×Albumin). OR represents

the multiplicative interaction term.To verify whether there is an interaction between COP and fluid

balance/albumin, a multivariate logistic regression model incorporating interaction terms was

constructed: aki ~ cop_post * fluid_balance + age + gender + weight + rachs + cpb_time +

circ_arrest + Albumin. The significance of interaction terms was assessed using the Wald test, with

a P-value <0.05 indicating significant interaction.

Supplementary Table 10. Mediation Analysis: Fluid Balance and Albumin as Mediators

| **Analysis** | **Indirect.Effect..ACME.** | **Direct.Effect..ADE.** | **Total.Effect** | **Proportion.Mediated** |
| --- | --- | --- | --- | --- |
| Overall - Fluid Balance | -0.0008 (-0.0045, 0.0004) p=0.230 | 0.0070 (-0.0017, 0.0102) p=0.062 | 0.0063 (-0.0029, 0.0077) p=0.090 | -12.2% (p=0.308) |
| ≤6 months - Fluid Balance | -0.0019 (-0.0096, 0.0021) p=0.388 | 0.0096 (-0.0163, 0.0143) p=0.290 | 0.0078 (-0.0197, 0.0103) p=0.384 | -24.0% (p=0.660) |
| >6 months - Fluid Balance | -0.000001 (-0.0002, 0.0002) p=0.902 | **0.0010 (0.000006, 0.0036) p=0.010** | **0.0010 (0.000007, 0.0036) p=0.006** | -0.1% (p=0.908) |

**Table Note:** The Causal Mediation Analysis framework (Imai et al., 2010) was employed to

estimate indirect and direct effects using the following two models: Mediator variable model

(linear regression): fluid_balance ~ cop_post + age + gender + weight + rachs + cpb_time +

circ_arrest + Albumin; Outcome variable model (logistic regression): aki ~ cop_post +

fluid_balance + age + gender + weight + rachs + cpb_time + circ_arrest + Albumin. The effect

estimation employed a nonparametric Bootstrap method (with 1,000 repeated sampling) to

estimate the following effects and their 95% confidence intervals: Average Causal Mediation

Effect (ACME): The indirect effect of COP on AKI through fluid balance; Average Direct Effect

(ADE): The direct effect of COP on AKI after adjusting for fluid balance; Total Effect: The sum of

ACME and ADE; Mediated Proportion: The proportion of ACME in the total effect.Negative

proportion mediated indicates suppression effect.Bolded values indicate statistical significance (p

< 0.05)

**Supplementary Table 11. Model Performance for AKI Prediction**

| **Model** | **AUC (95% CI)** | **Sensitivity**  **(%)** | **Specificity**  **(%)** | **PPV**  **(%)** | **NPV**  **(%)** | **Accuracy**  **(%)** | **LR+** | **LR-** |
| --- | --- | --- | --- | --- | --- | --- | --- | --- |
| Model 1: COP only | 0.592 (0.508-0.676) | 68.4 | 48.6 | - | - | - | - | - |
| Model 2: Clinical variables | 0.701 (0.625-0.778) | - | - | - | - | - | - | - |
| Model 3: Clinical + COP | ****0.723 (0.649-0.797)**** | ****71.9**** | ****62.4**** | ****38.7**** | ****87.1**** | ****64.8**** | ****1.91**** | ****0.45**** |

**Table Note:** Model 2 sensitivity and specificity are not reported as the model was primarily used

for AUC comparison with Model 3. Model 3 optimal cutoff determined by Youden index (0.228).

Supplementary Table 12. Net Reclassification Improvement (NRI) After Adding COP to Clinical Model

|  | **n (%)** | **Estimate (95% CI)** | **p-value** |
| --- | --- | --- | --- |
| **Net Reclassification Improvement (NRI)** |  | 0.070 (-0.062 to 0.196) | 0.30 |
| NRI for events (AKI=1) |  | 0.035 (-0.078 to 0.155) | 0.54 |
| NRI for non-events (AKI=0) |  | 0.035 (-0.027 to 0.095) | 0.27 |
|  |  |  |  |
| **Reclassification among events (n=57)** |  |  |  |
| Upward reclassification | 7 (12.3%) | 0.123 (0.050 to 0.215) | <0.001 |
| Downward reclassification | 5 (8.8%) | 0.088 (0.019 to 0.164) | 0.02 |
| Net reclassification | +2 (3.5%) | 0.035 (-0.078 to 0.155) | 0.54 |
|  |  |  |  |
| **Reclassification among non-events (n=173)** |  |  |  |
| Upward reclassification | 12 (6.9%) | 0.069 (0.035 to 0.111) | <0.001 |
| Downward reclassification | 18 (10.4%) | 0.104 (0.063 to 0.148) | <0.001 |
| Net reclassification | +6 (3.5%) | 0.035 (-0.027 to 0.095) | 0.27 |

**Table Note：**NRI: Net Reclassification Improvement; CI: Confidence Interval.

Net reclassification = Upward - Downward. Positive values indicate improved classification.

**Supplementary Table 13. Age-Dependent Clinical Utility of COP: Decision Curve Analysis Results**

| **Age Group** | **n** | **AKI Rate** | **Threshold** | **Net Benefit**  **(Clinical)** | **Net Benefit**  **(Clinical + COP)** | **COP Incremental Value** | **Clinical Interpretation** |
| --- | --- | --- | --- | --- | --- | --- | --- |
| ****0-3 months**** | 52 | 30.7% | 0.15 | 0.1923 | 0.1957 | +0.0034 | Minimal added value |
| ****4-6 months**** | 74 | 28.4% | 0.15 | 0.1940 | 0.1963 | +0.0024 | Minimal added value |
| ****7-9 months**** | 49 | 22.5% | 0.15 | 0.1116 | 0.1116 | 0.0000 | No added value |
| ****10-12 months**** | 55 | 16.4% | 0.15 | ****-0.0032**** | ****0.0385**** | ****+0.0417**** | ****Substantial added value**** |

**Table Note:** At threshold probability 0.15, representing a clinically relevant risk level for

intervention.Age-dependent clinical utility: Decision curve analysis results: The decision curve

analysis (DCA) was used to evaluate the net benefit across different age groups after incorporating

postoperative colloid osmotic pressure (COP) into the clinical model. The clinical model included

age, sex, weight, RACHS-1 score, cardiopulmonary bypass (CPB) duration, circulatory arrest, and

fluid balance. A threshold probability of 0.15 represented the risk level for clinical intervention

(i.e., intervention was considered when predicted AKI risk exceeded 15%). Higher net benefit

values indicated better clinical utility of the model at this threshold. COP incremental value was

defined as the difference between the total model and clinical model net benefits. Results showed

that in infants aged 10–12 months, the clinical model itself had a negative net benefit (-0.0032),

suggesting that using the clinical model alone might lead to unnecessary interventions. However,

the net benefit increased to 0.0385 (incremental value + 0.0417) after adding COP, reaching a

clinically acceptable level. In other age groups, the incremental value of COP was minimal

(0.0000–+0.0034). n: Sample size; AKI: Acute kidney injury.

**Supplementary Table 14.Subgroup analysis result table.**

| **Analysis** | **OR.per.mmHg..95..CI.** | **P.value** |
| --- | --- | --- |
| Main analysis (fully adjusted) | 1.16 (1.04-1.28) | 0.004 |
| Low albumin subgroup (n=115) | 1.11 (0.91-1.37) | 0.3 |
| High albumin subgroup (n=115) | 1.14 (0.96-1.37) | 0.12 |
| Low fluid balance subgroup | 1.12 (0.95-1.35) | 0.186 |
| High fluid balance subgroup | 1.27 (1.00-1.64) | 0.06 |
| Age 0-3 months (n=52) | 1.06 (0.87-1.29) | 0.584 |
| Age 4-6 months (n=74) | 1.08 (0.86-1.36) | 0.522 |
| Age 7-9 months (n=49) | 1.01 (0.71-1.40) | 0.956 |
| Age 10-12 months (n=55) | 1.79 (1.09-3.64) | 0.048 |
| Stepwise adjustment (M1→M4) | 1.12 (0.99-1.26) → 1.12 (0.99-1.28) | - |
| Segmented regression threshold | 16 mmHg (clinical threshold) | - |

**Table Note:** Albumin and fluid-balance subgroup analyses, exploratory age-stratified analyses, sequential adjustment models, and the exploratory 16-mmHg COP cutoff analysis are presented as supportive analyses. The age-stratified logistic association reached statistical significance only in infants aged 10–12 months, whereas estimates in younger groups were imprecise. The 16-mmHg cutoff should not be interpreted as a validated clinical treatment threshold. Across analyses, effect estimates were generally directionally consistent, although precision varied. OR: odds ratio; CI: confidence interval.

**Supplementary Table 15. Sensitivity Analysis of Ultrafiltration and Peri-CPB Management Factors**

Panel A. Sensitivity analyses of the post-CPB COP–AKI association

| Analysis | n | AKI events | Adjusted OR per 1-mmHg COP | 95% CI | P value |
| --- | --- | --- | --- | --- | --- |
| Primary analysis | 230 | 57 | 1.16 | 1.04–1.28 | 0.004 |
| + Total UF volume (mL/kg) | 230 | 57 | 1.13 | 0.99–1.29 | 0.075 |
| + Preoperative albumin | 230 | 57 | 1.15 | 1.03–1.29 | 0.012 |
| Excluding preoperative albumin <35 g/L | 214 | 51 | 1.17 | 1.03–1.33 | 0.015 |
| Acyanotic infants only | 218 | 55 | 1.15 | 1.00–1.32 | 0.050 |

Panel B. Ultrafiltration-related regression analyses

| Analysis | Effect estimate | 95% CI | P value |
| --- | --- | --- | --- |
| CPB duration → total UF volume | 0.025 mL/kg per min | −0.022 to 0.073 | 0.294 |
| Total UF volume → post-CPB COP | 0.010 mmHg per mL/kg | −0.007 to 0.028 | 0.252 |
| Total UF volume in the expanded AKI model | OR 0.99 per mL/kg | 0.96–1.01 | 0.303 |

Point estimates remained similar in direction and magnitude after reviewer-requested adjustment or restriction, although confidence intervals widened in several analyses. These analyses should not be interpreted as proving absence of residual confounding.

Supplementary Table 16. Collinearity assessment and sensitivity analysis for cross-clamp time

Panel A. Correlation between CPB time and cross-clamp time

| **Statistical Quantity** | **Value** |
| --- | --- |
| Pearson correlation coefficient r | 0.927 |
| 95% CI | 0.906–0.943 |
| P value | <0.001 |

Panel B. Variance inflation factor (VIF) for model variables

| Variable | VIF | Collinearity Test |
| --- | --- | --- |
| Age_m | 2.51 | — |
| gender | 1.13 | — |
| weight_kg | 3.46 | — |
| PreCr | 1.05 | — |
| RACHS_1 | 1.39 | — |
| Shunt time_min | 9.04 | >5. There is severe multicollinearity. |
| Blocking time_min | 8.34 | >5. There is severe multicollinearity. |
| Procedure: Stop Circulation | 1.27 | — |
| fluid_balance_per_kg | 1.12 | — |
| albumin_per_kg | 2.22 | — |

Panel C. Sensitivity analysis: cross-clamp time substituting CPB time in the primary model

| Model | COPOR | 95%CI | P **Value** |
| --- | --- | --- | --- |
| Main model (including CPB duration) | 1.16 | 1.04–1.28 | 0.004 |
| Alternative model (including blocking time) | 1.23 | 1.06–1.43 | 0.005 |

Table Note: VIF > 5 indicates problematic collinearity. CPB time and cross-clamp time were highly correlated (r = 0.927, VIF 9.04 and 8.34, respectively), precluding simultaneous inclusion in the primary model. The sensitivity analysis substituting cross-clamp time for CPB time yielded a directionally consistent COP estimate (OR 1.23, 95% CI 1.06–1.43, P=0.005), supporting the robustness of the main finding.

**Supplementary Table 17. Model specification sensitivity analysis for the COP–AKI association**

| **Model** | **Adjustments** | **OR per 1-mmHg COP** | **95% CI** | **P** |
| --- | --- | --- | --- | --- |
| Primary model | Age, sex, weight, RACHS-1, CPB time, circulatory arrest, fluid balance, albumin dose | 1.16 | 1.04–1.28 | 0.004 |
| Model 1 | Age, sex, weight, baseline creatinine, RACHS-1, CPB time, circulatory arrest | 1.21 | 1.05–1.40 | 0.009 |
| Model 2 | Model 1 + fluid balance + albumin dose | 1.24 | 1.07–1.44 | 0.005 |
| Model 3 | Model 1 + UF volume (mL/kg) + albumin dose | 1.22 | 1.05–1.41 | 0.009 |

Supplementary Table 18. Sample density by age and COP region

| **Age Group** | **COP Range** | **N** | **AKI Events** | **AKI Rate** | **COP Median (IQR)** |
| --- | --- | --- | --- | --- | --- |
| ≤3 months | 10–14 mmHg | 10 | 2 | 20.0% | 12.2 (12.0–12.5) |
| ≤3 months | 14–16 mmHg | 12 | 3 | 25.0% | 14.9 (14.4–15.1) |
| ≤3 months | 16–18 mmHg | 13 | 6 | 46.2% | 16.8 (16.7–17.1) |
| ≤3 months | 18–22 mmHg | 17 | 5 | 29.4% | 18.9 (18.3–20.5) |
| 4–6 months | 10–14 mmHg | 14 | 4 | 28.6% | 12.6 (12.2–13.4) |
| 4–6 months | 14–16 mmHg | 23 | 5 | 21.7% | 15.3 (14.9–15.7) |
| 4–6 months | 16–18 mmHg | 23 | 6 | 26.1% | 16.6 (16.3–17.1) |
| 4–6 months | 18–22 mmHg | 14 | 6 | 42.9% | 19.2 (18.6–19.8) |
| 7–9 months | 10–14 mmHg | 10 | 2 | 20.0% | 13.1 (12.5–13.6) |
| 7–9 months | 14–16 mmHg | 22 | 5 | 22.7% | 15.1 (14.8–15.5) |
| 7–9 months | 16–18 mmHg | 10 | 2 | 20.0% | 17.2 (16.7–17.6) |
| 7–9 months | 18–22 mmHg | 7 | 2 | 28.6% | 18.1 (18.0–20.0) |
| 10–12 months | 10–14 mmHg | 15 | 2 | 13.3% | 12.2 (12.0–12.8) |
| 10–12 months | 14–16 mmHg | 19 | 2 | 10.5% | 15.1 (14.5–15.5) |
| 10–12 months | 16–18 mmHg | 14 | 2 | 14.3% | 16.6 (16.2–17.4) |
| 10–12 months | 18–22 mmHg | 7 | 3 | 42.9% | 19.8 (18.2–20.1) |

Table Note: Age and COP strata correspond to the regions displayed in Figure 3. COP ranges: 10–14, 14–16, 16–18, 18–22 mmHg. Data are observed values from the study cohort (n=230). All regions contain at least 7 observations; no region is sparse. AKI, acute kidney injury; COP, colloid osmotic pressure; IQR, interquartile range.

**Supplementary Figure 1.** Directed acyclic graph of the temporal and causal framework for post-CPB COP and AKI

**Figure caption:** Solid arrows indicate assumed temporal relationships. The red arrow highlights the primary exposure–outcome association. Dashed boxes indicate unmeasured perfusion factors (hematocrit, oxygen delivery, mean arterial pressure, vasoactive exposure) that may influence both intraoperative management decisions and post-CPB COP. Variables in the post-exposure period (ICU fluids, diuretics, vasoactive support) were not included in the primary model. Importantly, the study did not have a true preoperative baseline COP prior to IV fluid administration; the earliest available patient-level COP measurement was obtained after heparinization and perioperative IV therapy had commenced, and is therefore not represented as a baseline node in this framework.

**Supplementary Figure 2.Age-specific dose-response curve of postoperative COP and AKI**

**risk**

Figure caption: The relationship between postoperative colloid osmotic pressure (COP) and acute kidney injury (AKI) risk at representative ages (3, 6, 9, and 12 months) was predicted from the generalized additive model (GAM). The model adjusted for fluid balance, gender,

body weight, RACHS-1 score, cardiopulmonary bypass (CPB) duration, and circulatory arrest.

Colored lines represent predicted risks for each age group, with light-colored shaded areas

indicating 95% confidence intervals. Vertical dashed lines mark the clinically relevant threshold

of 16 mmHg. The figure demonstrates that the effect of COP on AKI risk increases with age:

infants at 3 months (blue line) exhibit the highest baseline risk but the steepest slope, while

infants at 12 months (red line) have the lowest baseline risk but the steepest slope. When COP

rises from 10 mmHg to 22 mmHg, AKI risk increases from approximately 22% to 42% in

3-month-old infants and from about 1% to 17% in 12-month-old infants. These findings suggest

that although older infants have lower baseline risks, they exhibit greater relative sensitivity to

elevated COP.

**Supplementary Figure 3. Age–COP Joint Effect Heatmap**

**Figure caption:** A heatmap depicting the combined effect of age and postoperative colloid osmotic pressure (COP) on the risk of acute kidney injury (AKI), constructed using the Generalized Additive Model (GAM). The model accounted for covariates including gender, body weight, RACHS-1 score, CPB duration, circuit discontinuation, fluid balance, and albumin dose. The color scale ranges from light to dark, representing increasing predictive risk. Green dashed boxes denote the low-risk zone, yellow dashed boxes denote the intermediate-risk zone, and red dashed boxes denote the high-risk zone; these boxes serve as a visual aid for interpreting risk rather than constituting validated clinical thresholds.

**Supplementary Figure 4. Age-stratified mediating path diagram: Comparison of liquid balance mediating effects between infants ≤6 months and>6 months.**

**Figure caption:** The left panel shows infants ≤6 months (n=126), and the right panel shows infants>6 months (n=104). The path diagram illustrates the relationships between postoperative colloid osmotic pressure (COP), fluid balance, and acute kidney injury (AKI) across age groups. Path coefficients represent standardized regression coefficients, with solid arrows indicating significant paths, dashed arrows indicating non-significant paths, and bold red arrows indicating highly significant direct effects. In the ≤6-month group, neither the indirect effect (ACME-0.0019, P=0.39) nor the direct effect (ADE 0.0097, P=0.29) was significant. In the>6-month group, the indirect effect was not significant (ACME ≈0, P=0.90), but the direct effect was highly significant (ADE 0.0010,95% CI 0.000006–0.0036, P=0.01), suggesting that COP primarily affects AKI through direct nephrotoxic pathways. The model adjusted for gender, cardiopulmonary bypass (CPB) duration, and fluid balance. P<0.05, P<0.01, P<0.001.

**Supplementary Figure 5. ROC curves for three prediction models.**

**Figure caption:** Based on the linear relationship indicated by RCS analysis, a traditional Logistic regression model was employed to quantify the effect of each unit increase in COP. Three Logistic regression models were constructed for comparison: Model 1 (containing only COP): aki ~ cop_post; Model 2 (clinical model): aki ~ age + gender + weight + rachs + cpb_time + circ_arrest + Albumin dose + fluid_balance; Model 3 (full model): aki ~ cop_post + age + gender + weight + rachs + cpb_time + circ_arrest + Albumin dose + fluid_balance. The diagonal line served as the reference line (AUC=0.5).

**Supplementary Figure 6. Calibration curve for the full model**

**Figure caption:** Grouped by decimal place of predicted probabilities, demonstrating the consistency between model predicted probabilities and actual observed probabilities. The size of points represents the sample size of each group. The diagonal line (dashed) indicates ideal

calibration (predicted = observed), while the red solid line represents the local weighted regression smoothing line, with the light red area indicating the 95% confidence interval. The

Hosmer-Lemeshow test yielded a P-value of 0.638, indicating good model calibration.

**Supplementary Figure 7. Population-wide decision curve**

**Figure caption:** Population-wide analysis revealed that the net benefit after COP inclusion

showed only marginal improvement within a narrow threshold range (0.15–0.30).

Net benefit curves were plotted based on decision curve analysis (DCA) for clinical models

(clinical variables) across different age groups and the full model (clinical variables + COP). The

X-axis represents threshold probabilities, indicating the risk level considered for intervention; the

Y-axis represents net benefit, with higher values indicating better clinical utility of the model. The

diagonal line (gray solid line) represents the "full intervention" strategy, while the horizontal line

(gray dashed line) represents the "full non-intervention" strategy.

**Supplementary Figure 8. Age-stratified decision curve**

**Figure caption:** Age-stratified decision curve analysis (DCA) plots the net benefit curves of

clinical models (clinical variables) versus the full model (clinical variables + COP) across

different age groups. Results indicate that for infants aged 0-3 months (n=52): the incremental net

benefit after adding COP was minimal (+0.0034). For infants aged 4-6 months (n=74): the

incremental net benefit after adding COP was minimal (+0.0024). Infants aged 7-9 months (n=49): No improvement in net benefit was observed after adding COP (0.0000). Infants aged 10-12 months (n=55): The clinical model itself showed negative net benefit, suggesting potential for unnecessary intervention; however, the net benefit significantly increased (+0.0417) after adding COP, reaching a clinically acceptable level. The clinical value of COP is primarily demonstrated in the 10-12 month infant population, with its core function being to identify high-risk individuals within low baseline risk groups. These findings are highly consistent with the age-stratified analysis, which revealed COP effects observed only in older infants.

**Supplementary Figure 9. Forest plot of sensitivity analysis**

**Figure caption:** Forest plot illustrating the association between postoperative colloid osmotic

pressure (COP) and acute kidney injury (AKI) in principal analysis and various sensitivity

analyses. Each box represents the odds ratio (OR), with horizontal lines indicating 95%

confidence intervals (CI). Vertical dashed lines mark OR=1 (no effect). Box size reflects the

relative sample size in each analysis. Principal analysis (fully adjusted model): OR 1.16 (95% CI

1.04–1.28, P=0.004).

**Supplementary Figure 10. Comparison of standardized mean difference (SMD) of covariates**

**before and after IPTW weighting**

**Figure caption:** Standardized mean difference (SMD) of each covariate between the high COP

group (>16 mmHg) and low COP group (≤16 mmHg) before and after inverse probability

weighting (IPTW). An SMD <0.1 indicates good intergroup balance. Results showed that

covariates such as age and weight exhibited significant imbalance (SMD> 0.1) before weighting;

after weighting, all covariate SMDs decreased below 0.1, demonstrating that IPTW effectively

controlled for confounding factors. Covariates included: age, sex, weight, RACHS-1 score, CPB

duration, circulatory arrest, and fluid balance. SMD: Standardized mean difference; IPTW: Inverse

Probability Weighting; COP: Colloid osmotic pressure.

**Supplementary Figure 11. Postoperative COP quartiles and AKI risk**

**Figure caption:** Bar chart of AKI incidence rates by quartiles: Postoperative colloid osmotic

pressure (COP) was divided into four groups according to quartiles: Q1 (<14.2 mmHg), Q2 (14.2

15.7 mmHg), Q3 (15.7–17.4 mmHg), and Q4 (>17.4 mmHg). The incidence rates of acute kidney

injury (AKI) showed a gradient increase across groups, with values of 16.9%,21.1%,30.0%, and

31.5%, respectively. The dashed line represents the trend line. The Cochran-Armitage trend test (P = 0.039) confirmed a significant dose-response relationship between AKI risk and increasing COP levels.
